# An Experimental Evaluation of Toxicity Effects of Sodium Chloride on Oviposition, Hatching and Larval Development of *Aedes albopictus*

**DOI:** 10.3390/pathogens11020262

**Published:** 2022-02-18

**Authors:** Xiang Guo, Siyun Zhou, Jing Wu, Xiaoqing Zhang, Yuji Wang, Zixuan Li, Xiao-Guang Chen, Xiaohong Zhou

**Affiliations:** 1 Institute of Tropical Medicine, School of Public Health, Southern Medical University, Guangzhou 510515, China; guoxiang199399@163.com (X.G.); zhousy2326@163.com (S.Z.); wuzzz9800@163.com (J.W.); xiaoqingz1996@163.com (X.Z.); sj949643076@163.com (Y.W.); l_599x@163.com (Z.L.); xgchen@smu.edu.cn (X.-G.C.); 2 Key Laboratory of Prevention and Control for Emerging Infectious Diseases of Guangdong Higher Institutes, Guangdong Provincial Key Laboratory of Tropical Disease Research, Department of Pathogen Biology, School of Public Health, Southern Medical University, Guangzhou 510515, China

**Keywords:** *Aedes albopictus*, habitat, Sodium Chloride (NaCl), dengue, 50% lethal concentration

## Abstract

Dengue virus, one of the most important mosquito-borne viruses, has shown a sharp upward trend, spreading around the world in recent years. Control of vectors *Aedes aegypti* and *Ae. albopictus* remains crucial for blocking dengue transmission. The lethal ovitrap (LO) is one of the cost-effective traps based on the classic “lure and kill” strategy, and finding a proper long-lasting effective toxin is key to achieving the desired effect. The concentration of inorganic salts of habitat environment plays a strong role in affecting oviposition, hatching, and development of mosquitoes, but the potential insecticide activity of Sodium Chloride (NaCl) in habitat water as well as LO still lacks research. In this study, we carried out laboratory experiments to systematically explore the effects of different concentrations of NaCl solutions on oviposition, egg hatching, and larval development of *Ae. albopictus*. Consequently, *Ae. albopictus* was found to prefer freshwater to lay eggs; whereas 48.8 ± 2.6% eggs were laid in freshwater and 20% in ≥1.0% brackish water, few eggs were laid in 3.0% NaCl solution. Compared with egg hatching, larval development of *Ae. albopictus* presented a higher sensibility to NaCl concentration. The mortality of the 3rd–4th larvae in 1.0% NaCl solution was 83.8 ± 8.7%, while in 3.0% it reached 100%. Considering the cumulative effect of NaCl, when NaCl concentration was ≥1.0%, no eggs could successfully develop into adults. These data suggested that NaCl solutions with a concentration ≥1.0% can be used as an effective cheap insecticide for *Ae. albopictus* in subtropical inland aquatic habitats, and also as the “kill” toxin in LOs. Meanwhile, the concentration range from 0 to 2.0% of NaCl solution has the potential to be used as the “lure” in LOs. The technological processes of how to use NaCl as insecticide or in LOs still needs further in-depth exploration.

## 1. Introduction

The prevalence of dengue fever (DF) has shown an upward trend worldwide in recent years. It is estimated that 390 million dengue infections occur annually, of which 96 million have clinical symptoms [1,2]. In China, since the re-emergence of dengue in Foshan, Guangdong in 1978, numerous cases have occurred every year, arousing the widespread concern of the government and the public. Especially notable is the 2014 large-scale dengue epidemic that broke out mainly in Guangdong province, with 45,224 cases being reported, an incidence rate of 47.3 per 100,000 people [3]. As there are no specific anti-viral drugs or vaccines, controlling and eliminating breeding places of vector *Aedes albopictus* and *Ae. aegypti* remain important in the suppression of mosquito populations and the prevention of dengue. However, traditional approaches including space spraying of insecticides, environmental management, and larvivorous fish in controlling mosquito breeding habitats, have become insufficient and pose an urgent demand that alternative tools be developed and current strategies optimised [4].

“Lure and kill” is a classic strategy to design tools that utilise the ecological behaviours for vector *Aedes* control. With the help of artificial odor molecules, UV light, sex pheromone, and fit habitat, varied lures are conducted based on host-seeking, phototaxis, mating, and oviposition behaviour of vector mosquitoes [5,6]. Physical structure, suction of power fan, pesticide-releasing, biological pesticides such as BtiBlock and *Metarhizium* spp. are common methods of killing mature and immature stages of vector mosquito [7,8,9].

Lethal ovitrap (LO) is an essential type of “lure and kill” trap, which lures gravid female mosquitoes and kills their progeny, modified based on a traditional ovitrap (artificial habitat) [10]. LO is cost-effective, but there is still room for improving the flexibility in design and reducing the cost. One factor key to LO achieving its desired effect is finding a proper long-lasting effective toxin. In order to avoid the use of chemical insecticides which lead to residual toxicity, biomagnifications, and unknown harmful impacts, the development and application of bio-pesticides as vector control agents have attracted increasing interest in recent years. The potential agents, include bacteria (such as *Bacillus thuringiensis* subspecies Bt. Israelensis), virus (such as denso-virus), and fungi (such as *Metarhizium* spp.), etc, whose use in LO has been investigated [5,8,11,12]. At present, due to the reproduction difficulties, complex wild environment or limited effective time of these bio-control agents, the large-scale application of most of them still needs further exploration.

The concentration of inorganic salts in environmental aquatic habitats plays a crucial role in affecting oviposition, hatch, and development of mosquitoes, which can further influence the abundance and distribution of their aquatic stages. The potential effect of Sodium Chloride (NaCl) in water habitats, as the most common inorganic salt, is worth being explored. Larvae and pupae of *Ae. albopictus* and *Ae. aegypti* have long been considered to be freshwater-restricted. Reduction in oviposition above 0.5% NaCl showed that NaCl can be an oviposition repellent for gravid *Ae. albopictus* females [13]. In order to kill larvae of mosquitoes in cities, in Japan adding salt in tires and other potential habitats is recom-mended, and in Hong Kong and the Philippines sea water is diverted for drainage. However, a recent experiment showed that *Aedes* female adults could lay eggs in up to 3% NaCl concentration, and field-observed preimaginal stages could be found in brackish water (0.2–1.5%) in the coastal areas of the Philippines, Sri Lanka, and Brunei, which suggested the possible salinity-tolerance of *Aedes* [14,15,16].

Therefore, an in-depth understanding of the ecological effects of a wide range of concentrations of NaCl in vector *Aedes* mosquitoes’ environmental habitats still need to be considered. In this study, we preliminarily investigated the NaCl concentration in aquatic habitats in the city of Guangzhou, China. Next, we carried out laboratory experiments to systematically explore the toxicity effects of NaCl solution with different concentrations on oviposition, egg hatching, and larval development of *Ae. albopictus*, which will facilitate the development of relative strategies for the control of *Aedes* mosquitoes.

## 2. Results

### 2.1. Aquatic Habitats and Their NaCl Concentrations in Urban Environments

A total of 32 habitats in inland areas of Guangzhou, including a thrown-away lunchbox, a flowerpot, drainage ditches, impounded surface water, a discarded washing table, and tree holes, were analysed. All water samples of the aquatic habitats showed very low NaCl concentration (<0.05%, Salinometer WS-200 showed 0).

### 2.2. Toxicity Effects of NaCl Concentrations on Oviposition

**Figure 1:**
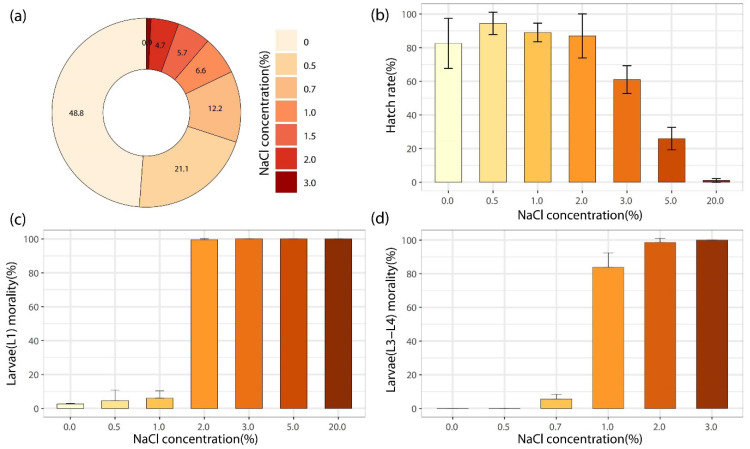
Experimental evaluation for competitive oviposition, hatching, and larval toxicity of *Ae. albopictus* under NaCl solution with different concentrations in lab. (**a**) The competitive oviposition rate in individual oviposition cups containing different NaCl concentrations. (**b**) The effect of NaCl concentrations on egg hatching. (**c**) The effect of different NaCl concentrations on development of 1st larvae of *Ae. albopictus*. (**d**) The effect of different NaCl concentrations on development of 3rd–4th larvae of *Ae. Albopictus*.

In total, the oviposition egg numbers of *Ae. albopictus* were 4551, 6366, and 6485 for three biological repetitions, and the laying egg number of each female was 39.6, 38.1, and 38.1, respectively. The efficiency of NaCl solutions with different concentrations (0, 0.5%, 0.7%, 1.0%, 1.5%, 2.0% and 3.0%) on egg induction was evaluated by comparative oviposition rate (COR), with 48.8 ± 2.6, 21.1 ± 1.7, 12.2 ± 3.3, 6.6 ± 1.2, 5.7 ± 1.7, 4.7 ± 1.1 and 0.9 ± 0.8, respectively. More than 80% of eggs were laid in NaCl concentrations with a range of 0–0.7%, and there was an obvious downward trend when concentrations increased. The amount of eggs attracted by the control group was about twice of that at 0.5% NaCl and about four times of that at 0.7% NaCl (Figure 1a). The results of ordinary one-way ANOVA showed that the differences between the control group and each experimental group were statistically significant (*F* = 218.5, *p* < 0.0001) (Figure 1a).

### 2.3. Toxicity Effects of NaCl Concentrations on Egg Hatching

One day after mature and fresh mosquito eggs were immersed in NaCl solution with different concentrations (0, 0.5%, 1.0%, 2.0%, 3.0%, 5.0% and 20.0%), the hatching rate (HR) of eggs at the tenth day reached 82.5 ± 14.9, 94.5 ± 6.6, 89.0 ± 5.5, 87.0 ± 13.1, 61.0 ± 8.3, 25.9 ± 6.7 and 1.1 ± 1.1, respectively (Figure 1b). It showed no statistically significant differences between HR at 0, 0.5%, 1.0% and 2.0% NaCl (ordinary one-way ANOVA chi-square test, *p* > 0.9999) (Figure 1b). However, when the NaCl concentration reached 2.0%, HR dropped rapidly with increased salt concentration, and almost no hatch occurred at 20.0% NaCl (Figure 1b).

### 2.4. Toxicity Effects of NaCl Concentrations on Larval Development

NaCl concentrations can significantly affect the development of the 1st and 3rd–4th instar larvae of *Ae. albopictus* (Figure 1c,d). When NaCl concentration reached 1.0%, the 1st instar larvae were observed inactive, and the 3rd–4th instar larvae began to die within 1–2 days, with the total mortality rate (MR) exceeding 80% (83.8 ± 8.7%). Meanwhile, if NaCl concentrations ≥2.0%, the mortality of 1st instar larvae presented 99.6 ± 0.7% within 24 h after hatching. If the concentration reached 3.0%, the 3rd–4th instar larvae died at 2 h and the mortality could reach 100% within 24 h. PROBIT analysis showed the 24 h 50% lethal concentrations (LC50) and 95% lethal concentrations (LC95) of NaCl solution on the 1st and 3rd–4th instar larvae of *Ae. albopictus* in Table 1.

### 2.5. The Cumulative Toxicity Effects of NaCl Concentrations on a Whole Life Cycle

**Figure 2:**
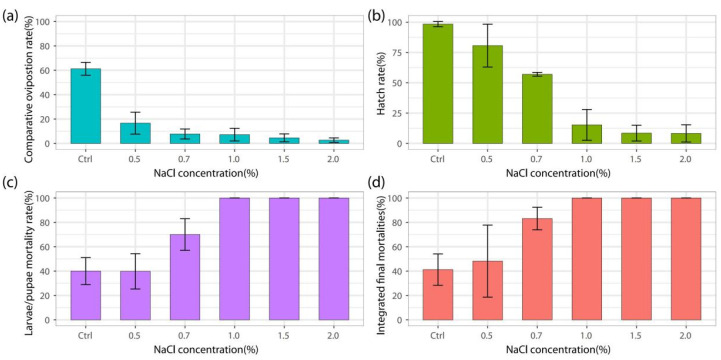
The cumulative toxicity effects that NaCl concentrations showed on oviposition: (**a**), egg laying (**b**), larval development (**c**), and the integrated final mortalities (**d**) of *Ae.* *albopictus*.

The cumulative toxicity effects of different NaCl concentrations (0, 0.5%, 0.7%, 1.0%, 1.5% and 2.0%) on oviposition, egg hatching, and larval development of *Ae. Albopictus* were continuously observed, and the integrated final mortalities (IFM) were thereby assessed with 41.2 ± 12.9, 48.2 ± 29.6, 83.1 ± 9.3, 100., 100, and 100, respectively (Figure 2). We found that the IFM of *Ae. albopictus* in 0.7% NaCl was higher than that in the control group, and when NaCl concentrations exceeded 1.0%, no eggs could successfully develop into adults (Figure 2).

## 3. Discussion

We found *Ae. albopictus* eggs in NaCl solutions with different concentrations from 0 to 3.0%. However, while 48.8 ± 2.6% eggs were laid in freshwater and 20% in ≥1.0% brackish water, few eggs were observed when NaCl concentration reached 3.0%. These data indicate that *Ae. albopictus* mosquitoes have a preference for freshwater to lay eggs, in accordance with previous studies [13,17]. Therefore, freshwater is an effective “lure” in LOs for attracting oviposition of *Ae. albopictus*, while NaCl solutions with concentrations ≤2.0% might be used as a “lure” in LOs under certain circumstances. As mosquitoes approach an oviposition site, they can use site-specific olfactory cues evaluated with antennal, labrum, and tarsal receptors as short-range signals for continuously perceiving the quality of the site [18]. In the assessment of an oviposition site with NaCl solutions of different concentrations, the related olfactory cues and molecular mechanism are still unclear [13].

Larvae rather than eggs of *Ae. albopictus* showed high sensibility to the increase of NaCl concentration. The hatching rate among NaCl concentrations with 0, 0.5%, 1.0%, and 2.0% had no statistically significant differences, as all of them could reach 80%. However, the mortality of the 3rd–4th larvae in 1.0% NaCl was 83.8 ± 8.7%, while in 2.0% NaCl could reach 100%. These findings indicated that the 3rd–4th instar larvae could be more susceptible to NaCl concentrations, which might be the cumulative toxicity effects attributed to longer exposure to the NaCl solution. Our experiments showed that LC95 of NaCl concentration on the 3rd–4th instar larvae was 11.4 g/L (1.14%), similar to previous studies [19]. Furthermore, taking the cumulative toxicity effects on a whole life cycle of *Ae. albopictus* into consideration, if NaCl concentrations ≥1.0%, no egg completes a full life cycle. Therefore, NaCl solutions with concentrations ≥1.0% can be used as an effective cheap insecticide for *Ae. albopictus* and the “kill” toxin in LOs as well.

In recent decades, the presence of climate warming, unplanned urbanisation, and increasing population mobility has led to the continued expansion of *Ae. albopictus* populations more along the fringes of their distribution, which has further shaped their ecological characteristics and formed their behaviour pattern [20,21,22]. The concept of integrated vector management and its efficient implementation are crucial to achieving sustainable vector management. However, the cost of vector control is still expensive for communities, cities, and even national governments, especially in most developing countries. The present systematic evaluation of toxicity effects of NaCl solution with different concentrations indicates that ≥1.0% NaCl has toxic effects on *Ae. albopictus* which can be utilised as a cheap environmentally friendly insecticide to control the vector populations. Thus, cheap methods such as the use of seawater and NaCl solutions with concentrations ≥1% are worth adopting for vector management in a wide range of application scenarios.

In previous practices in Hong Kong and the Philippines, seawater has been introduced into drainage systems for controlling larvae of mosquitoes. Here we would take the urban management of Guangzhou as an example and consider how to apply NaCl solutions with concentrations ≥1.0% for population suppression of *Ae. albopictus*. Guangzhou is located in the subtropical region of southern China. The residents in Guangzhou like to grow flowers and plants indoors or outdoors, or in public places, where flower pots have been investigated as one of the most important aquatic habitats of *Ae. albopictus*. The preliminary investigation result showed <0.05% NaCl concentration in natural aquatic habitats in the urban region of Guangzhou. Therefore, we suggest that the residents can easily dissolve edible salt in flower plots or other aquatic habitats for killing *Aedes* larvae, keeping NaCl concentrations ≥1.0%. For indoor use, 1.0% NaCl is suitable enough. Taking into consideration water evaporation and rainfall in outdoors environments, we recommend putting a little more salt in the corresponding outdoor water container, or adding appropriate amounts according to the situation, in order to maintain an ideal NaCl concentration. Therefore, The sentence is revised as follow: It is valuable and urgent to establish an effective scientific communication and support system for the sustainable control of vectors and the effective population suppression of *Aedes*. In Guangzhou, a megacity of 18.7 million people, each resident needs to know how to manage the aquatic habitats of *Ae. albopictus* through simple cheap ways, including the edible salt described above. It should be noted that the potentially different salinity tolerance of *Aedes* populations in coastal regions may affect the effective insecticide concentration of NaCl, which still needs further investigation.

Furthermore, the unpredictable cocktail effect needs further exploration of mixed NaCl salt with traditional chemical larvicide or novel biological larvicide. For example, NaCl concentration has a small but significant negative impact on the toxicity of *B. thuringiensis* toxin to *Aedes* larvae at NaCl concentration levels < 0.018 ppm where *Aedes* larvae can survive in the field [23]. However, temephos seemed to show different cross-interactions with NaCl concentration that, low salinity (1–3.5%) decreased the toxicity and high salinity (5.0%) increased the toxicity [24].

Therefore, the present study suggests that NaCl solutions with concentrations ≥1.0% can be used as effective cheap insecticides for *Ae. albopictus* in aquatic habitats. Meanwhile, NaCl solutions with concentrations ranging from 0 to 2.0% have the potential to be used as the “lure” in LOs. The technological processes of how to use NaCl—as insecticide, in LOs, or mixed with other larvicides—to help control vector mosquitoes and prevent dengue epidemics still needs further in-depth exploration.

## 4. Materials and Methods

### 4.1. Aquatic Habitats and Their NaCl Concentrations in Urban Environments

**Figure 3:**
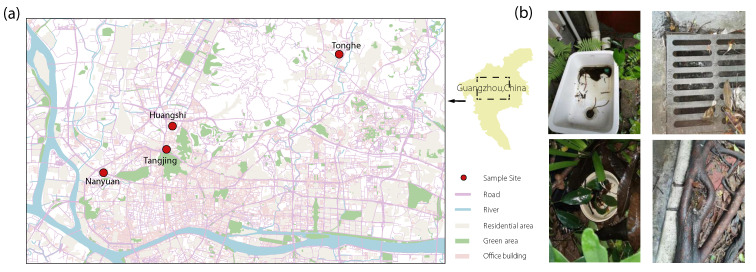
Sampling sites (**a**) and aquatic habitat environments (**b**) in urban region of Guangzhou. Red circles in (**a**) represent the four sample sites (Tonghe, Huangshi, Tangjing and Nanyuan) in Guangzhou, China.

Aquatic habitats of *Ae. albopictus* were surveyed in four study sites (Tonghe, 23°11′13″ N, 113°19′38″ E; Huangshi, 23°12′34″ N, 113°16′40″ E; Tangjing, 23°10′25″ N, 113°15′51″ E; Nanyuan, 23°8′39″ N, 113°14′40″ E) in May 2020 (Figure 3). The larval sampling was conducted using a standard dipping method. Samples were transported to the laboratory, where they were reared until emergence for species identification. Frozen mosquitoes were placed on a piece of white filter paper in a petri dish on a chill table, and the species was identified morphologically using taxonomic keys or based on DNA barcoding [25]. About 15 mL of habitat water was sampled at the center of each habitat using a 50 mL EP tube, then transported to the laboratory within 4 h. The NaCl concentration of each habitat water sample was detected using the Salinometer WS-200 (Scionix, China).

### 4.2. Mosquitoes

The subtropical inland population of *Ae. albopictus* was collected from the field in inland areas of Guangzhou in 2014 and reared in our laboratory [26]. Mosquitoes were maintained in bioclimatic insectary at 25 °C ± 2 °C, 60 % ± 20% relative humidity, and a light:dark photocycle of 16 h:8 h. The deionised breeding water used in the laboratory was salt-free, just like those of the wild breeding sites described above. The breeding process was as follow: eggs were incubated in deionised water with yeast powder (0.25 g/L water); larvae were fed with a combination of yeast powder and turtle food; pupae were put into 250 mL cups and transferred to a screen cage (22 cm × 22 cm × 34 cm); emerged adults were provided with a cotton pad soaked with 10% glucose solution; female adults (4–6 days post-eclosion) were starved for 24 h and then allowed to take blood for 8 h by feeding on an anaesthetic Kunming mouse (Experimental Animal Center of Southern Medical University). Three biological repetitions were prepared for the following explorations of the toxicity effects of NaCl solutions on oviposition, hatching, and larval development of *Aedes albopictus.* Each biological repetition of egg, larvae, or adult was reared in an independent cage.

### 4.3. The Effects of NaCl Concentrations on Oviposition

**Figure 4:**
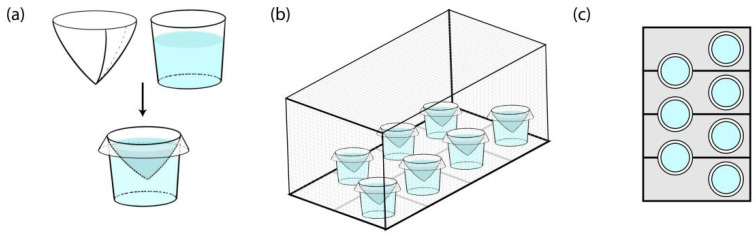
Cage and oviposition cups set in competitive oviposition experiment. (**a**) Schematic diagram showed oviposition cups set. Schematic diagram of side view (**b**) and top view (**c**) showed oviposition cups positions in cage.

Two hundred *Ae. albopictus* adults (ratio of male to female about 1:1, 3 days after blood-meal, Table S1) were placed in a cage, along with 7 individual black oviposition cups, each containing an NaCl solution of a different concentration (0, 0.5%, 0.7%, 1.0%, 1.5%, 2.0%, and 3.0%) (Figure 4). The different concentration solutions were prepared by dissolving pure NaCl (GR, Aladdin, Shanghai, China) in deionised water. Deionised water was also prepared in the control group. The initial number of newly laid eggs on each filter paper was counted each 24 h for 3 days. The COR was calculated as follows: COR% = eggs in certain NaCl concentration/total eggs in screen cage × 100%.

### 4.4. The Effects of NaCl Concentrations on Egg Hatching

The 5-day-old *Ae. albopictus* eggs (*n* = 92–138) of the same generation selected as a group were put in a hard plastic cup (90 mL). Seven groups contained same yeast powder (0.25 g/L water), and 70 mL NaCl solutions of different concentrations (0, 0.5%, 1%, 2%, 3%, 10% and 20%) were set for egg hatching. The larvae were picked out by pipette every 24 h, and the number of dead and alive larvae at each NaCl concentra-tion was counted until there were no new larvae. The HR was calculated as follows: HR% = dead and living larvae/total eggs × 100%.

### 4.5. The Effects of NaCl Concentrations on Larval Development

Under the same feeding condition, the 1st and 3rd–4th instar larvae of the same generation in the same group were selected (*n* = 20–25). Each group was placed in NaCl solution with different concentrations of 0, 0.5%, 0.7%, 1%, 2% and 3%. Each day, larval death and pupation were observed at 0 h, 1 h, 2 h, 4 h, 8 h, and 24 h. If a larva could not escape the light touch from a plastic straw, it would be considered dead. Death and/or pupation of all the larvae were treated as the end point of observation. The larvae MR was calculated as follows: MR 24 h% = dead larvae/dead and living larvae × 100%.

### 4.6. The Cumulative Effects of NaCl Concentrations on a Whole Life Cycle

Aiming to test the cumulative effect of NaCl concentrations on oviposition, egg laying, and larval development, namely a whole life cycle, *Ae. Albopictus* mosquitos were reared from adults to next-generation adults in NaCl solutions with concentrations of 0, 0.5%, 0.7%, 1.0%, 1.5% and 2.0%, respectively. Six ovi-cups were randomly put in the screen cage for ten days, and every two days the solution and filter paper were renewed. Fresh eggs were kept at 25 °C ± 2 °C and 80% ± 5% relative humidity for five days to ensure the complete development of the embryos. We put eggs into a 500 mL white plastic bowl with a cover (there was a vent hole on the cover), and added 400 mL of the corresponding NaCl solution. Hatched larvae were softly transferred by using a plastic straw into another identical bowl and kept breeding. Emerged adults were collected by a mosquito aspirator and frozen at −4 °C for counting. During the rearing process, we updated water bodies and fed larvae in time, according to water turbidity, smell, the number of larvae and their growth status. IFM% = 1-next-generation adults/eggs × 100%.

### 4.7. Data Analysis

Statistical analyses were performed using SPSS 22.0 (IBM) and R (www.R-project.org) (accessed on 3 January 2021). The effect of different NaCl concentrations on the oviposition of *Ae. albopictus* was mainly determined by a one-way ANOVA, but if *p* > 0.05 in a variance homogeneity test, it was assessed using Least-Significant Difference (LSD). Meanwhile, the effects of different NaCl concentrations on egg hatch and larval development were inferred using Chi-Square Tests. LC50 and LC90 were estimated for 1st and 3rd–4th instar larvae using the PROBIT analysis. The significance level was 0.05.

## Figures and Tables

**Table 1:** Median and 95% lethal NaCl concentrations (LC; g/L) estimated for 1st instar larvae (within 24 h) and 3rd–4th instar larvae of *Ae. albopictus*.

Larval Stage	1st Instar (within 24 h)	3rd–4th Instar
LC50	12.026 (11.388–12.718)	8.742 (8.060–9.511)
LC95	13.995 (13.185–15.219)	11.412 (10.292 ± 14.187)
slope	24.973 ± 3.368	14.211 ± 3.013
χ^2^	1.842	0.398
*p*	0.466	0.792

Note: The data were expressed as 95% confidence interval and mean ± standard error. Pearson Chi-square (χ^2^) goodness-of-fit test showed *p* > 0.15, indicating that this model fitted data well.

## Data Availability

Data supporting the conclusions of this article are included within the article and its Supplementary Materials.

## Supplementary Materials

The following materials are available online at https://www.mdpi.com/article/10.3390/pathogens11020262/s1, Table S1: The total number of eggs laid, average number of eggs per female mosquito laid, and the ratios of female to male adults in three replicate cages.
