# Supplementary material for: An Experimental Evaluation of Toxicity Effects of Sodium Chloride on Oviposition, Hatching and Larval Development of Aedes albopictus
Source: Pathogens. 2022 Feb 18;11(2):262. doi: 10.3390/pathogens11020262 (PMC8878149; doi:10.3390/pathogens11020262)
Supplement: Supplementary file 1 [file pathogens-11-00262-s001.zip › pathogens-1564009-supplementary.pdf]

Table S1. The total number of eggs laid, average number of eggs per female mosquito laid , and the ratios of female to male adults in three replicate cage.

Replicate	Total *No.*	Female : Male	Average *No.*	*F*	*P*
A	4551	115:88	39.6	3.00	0.333
B	6366	167:44	38.1		
C	6485	170:56	38.1		

We set three replicates. Total No. denotes the total number of eggs laid in screen cage. Female : male denotes the ratios of female to male adults in each cages. Average No. Denotes the average number of eggs per female mosquito laid. Ordinary one-way ANOVA showed no significant difference between three replicates.
